# Self-Reported Health in Adolescents With Exercise-Induced Laryngeal Obstruction; A Cross-Sectional Study

**DOI:** 10.3389/fped.2021.617759

**Published:** 2021-07-08

**Authors:** Merete R. Benestad, Jorunn Drageset, Hege Clemm, Ola D. Røksund, Maria Vollsæter, Thomas Halvorsen, Mari Hysing, Bente J. Vederhus

**Affiliations:** ^1^The Faculty of Health and Social Sciences, Western Norway University of Applied Sciences, Bergen, Norway; ^2^Department of Pediatrics, Haukeland University Hospital, Bergen, Norway; ^3^Department of Global Public Health and Primary Care, University of Bergen, Bergen, Norway; ^4^Department of Clinical Science, Faculty of Medicine, University of Bergen, Bergen, Norway; ^5^Department of Otolaryngology/Head and Neck Surgery, Haukeland University Hospital, Bergen, Norway; ^6^Norwegian School of Sport Sciences, Oslo, Norway; ^7^Department of Psychosocial Science, Faculty of Psychology, University of Bergen, Bergen, Norway

**Keywords:** exercise-induced laryngeal obstruction, self-reported health, adolescents, breathing difficulties, self-efficacy

## Abstract

**Background:** Exercise-induced laryngeal obstruction (EILO) is common in young people with exertional breathing difficulties. Psychological characteristics have been proposed as underlying contributors; however, the evidence for this is limited.

**Objectives:** Describe self-reported health, self-efficacy, and anxiety symptoms in adolescents with EILO, and address possible associations with EILO subtypes and severity.

**Methods:** Cross-sectional study of 71/180 (39%) adolescents tested for EILO at Haukeland University Hospital during 2014–2016, age range 14–18 years. Validated questionnaires were used to assess general self-rated health, subjective health complaints (Health Behavior in School-aged Children-Symptom Check List; HBSC-SCL), general self-efficacy (GSE), and anxiety symptoms (SCARED). The outcomes were compared with normative data from comparable unselected populations.

**Results:** The HBSC-SCL items for somatic complaints revealed weekly or more often occurrence of headache in 42%, abdominal pain in 30%, backache in 31%, and dizziness in 32%. For psychological complaints, corresponding figures were 26% for feeling low, 43% for irritability or bad mood, 33% for feeling nervous, and 38% for sleep problems. Mean (range) GSE score was 3.13 (2.2–4.0), and reports suggesting anxiety symptoms were rare. The outcomes were in line with normative data from comparable unselected populations. Self-rated health, and scores obtained for HBSC-SCL, GSE, and SCARED were similarly distributed across EILO subtypes and severity.

**Conclusion:** Self-reported health, self-efficacy, and level of anxiety symptoms in adolescents with laryngoscopically confirmed EILO were similar to data obtained in comparable unselected populations, irrespective of EILO subtype and severity. The findings challenge the notion that pediatric EILO is causally related to psychological problems.

## Introduction

Exercise-related breathing problems caused by laryngeal airflow obstruction (EILO) are relatively common in otherwise healthy young individuals. Studies of unselected childhood and adolescent populations report prevalence rates of 5 and 7%, respectively (1, 2). Moreover, a recent large study of recreational runners revealed inspiratory wheeze in nearly 10%, suggesting that EILO still might be an under-recognized condition in the general population (3). This clinical scenario was previously seen as a form of vocal fold (cord) dysfunction (VCD), and therefore often labeled exercise induced paradoxical vocal fold (cord) motion (PVFM or PVCM). Better diagnostic tools have improved the understanding that vocal fold adduction *per se* might not represent an “inciting incident” in many patients (4, 5). Therefore, a task force appointed by the European Respiratory Society (ERS), the European Laryngological Society (ELS), and the American College of Chest Physician (ACCP) has proposed a taxonomy that rests on findings made by laryngoscopy (6, 7). The umbrella term “inducible laryngeal obstruction” (ILO) should be linked to a description of the predominant anatomical location of the obstruction; e.g., glottic or supraglottic or both. The inducer should be listed first, hence the acronym EILO for obstruction induced by exercise.

Historically, VCD carried strong psychological and even psychiatric connotations (8–10). Recent literature have questioned this notion, and suggested that the causality might be reverse; i.e., that potential psychological associations might in fact be *secondary* to the dyspnea and the sense of failure to control breathing while under pressure (11). Studies of asthma have suggested links between respiratory symptoms and psychological and/or emotional factors and/or coping mechanisms (12–16). However, the *direction* of these associations is not self-evident, and may vary between patients. Although certainly important, these issues have not been properly addressed in young people with EILO. Traditionally, treatment of EILO has focused on breathing advice, speech therapy and psychological support. In recent years, there has been a development toward more “mechanistic interventions,” such as inspiratory muscle training and even irreversible surgery (17–22). The role of psychological problems in this group of patients is therefore important to establish.

We hypothesized that a higher rate of psychological symptoms would be most prominent in patients with the most severe findings, or in patients with glottic EILO, as this subtype might resemble what used to be labeled VCD. Our aim was to study self-reported health, self-efficacy, and anxiety across EILO subtypes and severity, diagnosed and classified as proposed by the ERS/ELS/ACCP nomenclature. A control group was not included, but the outcome data were compared to normative data obtained from large groups of comparable individuals.

## Methods

### Ethical Considerations

The Regional Committee for Medical Research Ethics for Western Norway had approved the study before it was commenced, Protocol no. 2017/299. Informed written consent was obtained from all participants.

### Subjects Descriptions

In this cross-sectional study, all adolescents (age range 14–18 years) enrolled in the EILO registry at Haukeland University Hospital in Bergen, Norway, during 2014–2016 were invited to participate. A letter of invitation, a paper-based consent form, the questionnaires listed below, and a prepaid return envelope, were sent by post to the participants' home address.

The EILO registry enrolls patients referred for investigation of unexplained exercise related breathing problems and tested for EILO by completing a continuous laryngoscopy exercise (CLE) test, performed as described in detail previously (23). Asthma and/or exercise induced bronchial obstruction (EIB) do not preclude enrollment in this registry; however, if present adequate treatment should be instituted and the presenting symptoms should not resemble those of EIB. Great care is taken to exclude (to the extent possible) that asthma plays a significant role in the enrolled patients' exercise related respiratory problems. Untreated or under-treated asthma is considered contraindication for performing a CLE-test, but well-treated stable asthma is not, as the two conditions may co-exist. Any use of asthma medication is reported in the questionnaire filled in at enrollment.

**Figure 1:**
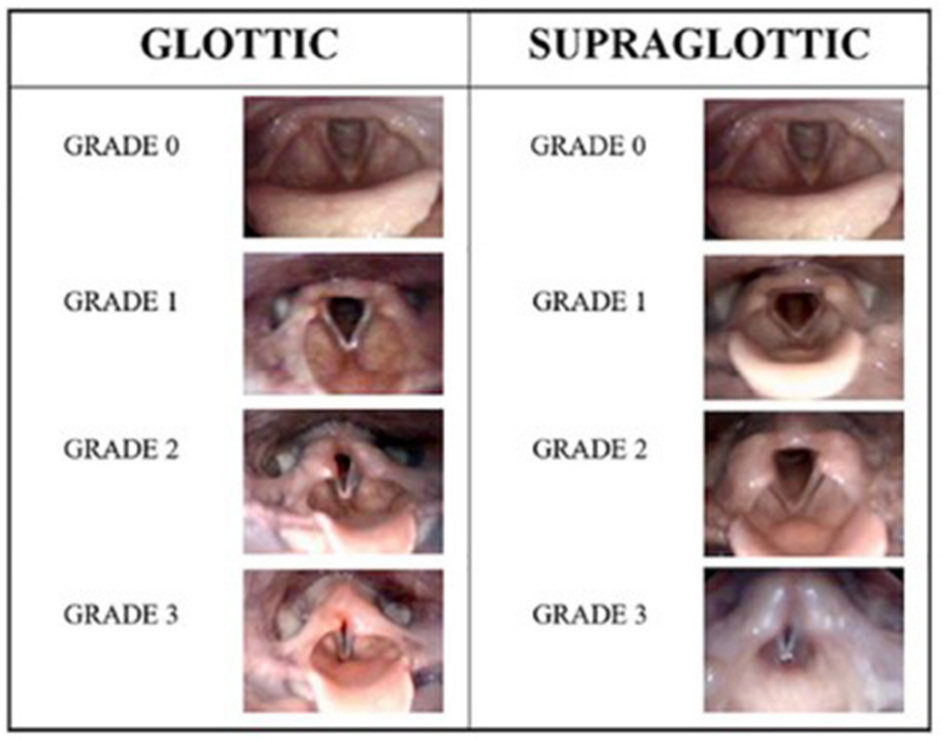
The human grading system according to Maat et al. (24) illustrated by endoscopic photographic images during exercise from the larynx showing the different grades of laryngeal obstruction at the glottic and supraglottic levels.

EILO was diagnosed according to obstruction in the larynx on two levels; glottic and supraglottic. EILO severity and subtype were scored according to published criteria (24). Only two highly experienced raters performed all scoring procedures. Both raters reviewed the videos retrospectively and individually, and all ratings were performed in one session, solving disagreements subsequently by consensus. No obstruction was classified as 0, mild as 1, moderate as 2 and severe as 3 (Figure 1) (24). For the purpose of this article, the patients were split into four subgroups based on their CLE scores obtained at maximum intensity exercise at the glottic and supraglottic laryngeal levels: 1 = no or mild obstruction at both levels; 2 = obstruction exceeding mild on glottic level only; 3 = obstruction exceeding mild on supraglottic level only; 4 = obstruction exceeding mild on both glottic and supraglottic level. For the purpose of this study, subgroup 1 was seen as normal; subgroup 2 as predominantly glottic EILO; subgroup 3 as predominantly supraglottic EILO; and subgroup 4 as severe EILO.

### Questionnaires

A general questionnaire was applied to obtain information on age, gender, health, medication use, and the level of parental education. Specific questions were asked about the participants' physical activities and sports, and how they performed their training, such as “*are you active in sport*,” “*what type of sport are you involved in*,” and “*how many times per month do you practice your sport*?.”

#### General Self-Rated Health

General self-rated health was assessed by the question “*would you say your health is*.” and then rated on a 4-point scale ranging from “*poor”* to “*excellent,”* and thereafter responses were assigned values from 1 to 4, respectively. The question has demonstrated good validity and reliability in adolescent populations (25, 26).

#### Subjective Health Complaints

The Health Behavior in School-aged Children – Symptom Check List (HBSC-SCL) assesses the occurrence of eight somatic and psychological symptoms. The participants were asked to rate the frequency experienced in the past 6 months with items divided into the psychological sub-score (feeling low/depressed, irritable/bad tempered, nervous, and sleeping difficulties) and the somatic sub-score (headache, abdominal pain, backache, and feeling dizzy). Each item was assessed on a 5-point response scale ranging from daily (4) to rarely/never (0). Two sub scores (0–16) and a total sum score (0–32) were calculated (27). Other studies have reported high reliability and validity for adolescents between 11 and 15 years (27, 28). We applied the translated and validated Norwegian version (28).

#### General Self-Efficacy (GSE)

GSE was measured using the 10-item scale based on Bandura's concept of self-efficacy. The scale is designed for subjects from 12 years of age and older (29). The GSE scale has demonstrated good psychometric properties (30, 31). A validated Norwegian version was used (31). The scale is measured on a 4-point Likert scale ranging from 1 (not true at all) to 4 (exactly true).

#### Anxiety

Anxiety symptoms were measured with the short version of the Screen for Child Anxiety Related Disorder (SCARED) developed for adolescents between 8 and 18 years (32). The scale measured five statements on a 3-point Likert scale ranging from not true (0) to often true (2). The items are summarized to calculate a total score ranging from 0 to 10. The short version has similar well-established psychometric properties as the original scale (32). No validation studies have been conducted on the Norwegian translation, but it has been used in different studies (33, 34).

### Statistical Analysis

Descriptive analyses were used to assess means and standard deviations (SD) of general health, anxiety, subjective health complaints and GSE, whereas demographic and clinical characteristics were reported as numbers and proportions. One-Way ANOVA was used to investigate if there were any differences between the four EILO sub-groups regarding general health, anxiety, subjective health complaint, and GSE. The criterion for statistical significance was *p*-value ≤ 0.05. All analyses were conducted using Statistical Package for the Social Sciences (SPSS) Version 24 for Windows.

## Results

### Participant Characteristics

There were 71/180 (39%) adolescents who returned the questionnaires, of whom 58 (82%) were females, and the mean (SD) age was 16 (1.4) years (Table 1). Background variables and EILO subgroups for the participants are displayed in Table 2. A larger fraction of non-responders than responders had been classified as normal (subgroup 1). In two of the responders, the quality of the CLE test did not allow sub-grouping, thus EILO could be classified in 69/71 participants. Few participants reported asthma or EIB, and there were no differences between the EILO subgroups (Table 3).

**Table 1:** Demographic and clinical characteristics among the participating adolescents with EILO^a^.

**Females (%)**	**58 (82)**
**Age; years (SD)**	**16 (1.4)**
**Number of training sessions per month; mean (range)**	**19 (12, 77)**
**Parents' educational level; college or higher; number (%)**	
** Mother**	**46 (66)**
** Father**	**38 (55)**

a *Exercise induced laryngeal obstruction*.

**Table 2:** Sub-groups of 69 participants^a^ with exercise induced laryngeal obstruction (EILO).

** 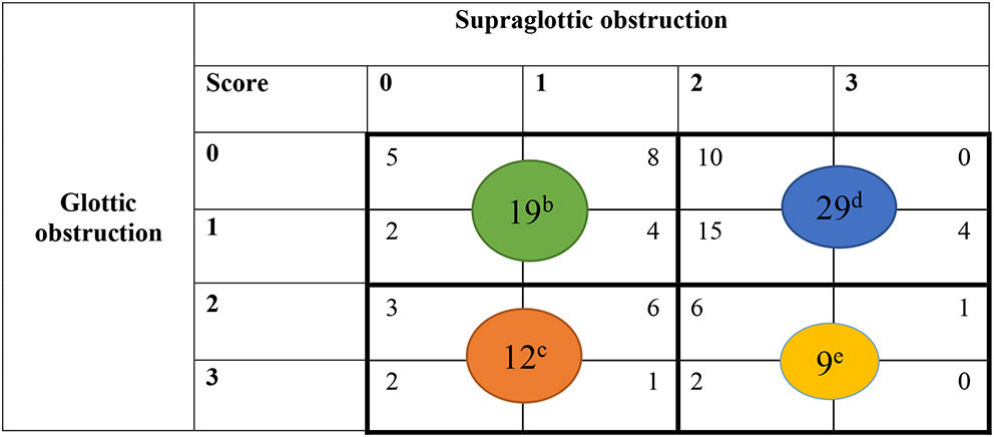 **

*EILO sub-groups*.

a *CLE scores missing for two responders*.

b *No or mild obstruction, both levels*.

c *Obstruction exceeding mild on glottic level only*.

d *Obstruction exceeding mild on supraglottic level only*.

e *Obstruction exceeding mild on glottic and supraglottic level*.

**Table 3:** Self-reported asthma characteristics, by EILO subgroups^a^.

	**Normal larynx (*n* = 15/19)^b^**	**Predominantly glottic (*n* = 11/12)^b^**	**Predominantlysupraglottic (*n* = 26/29)^b^**	**Combined glottic and supraglottic (*n* = 8/9)^b^**
Exercise induced bronchoconstriction *n*, %	1 (7)	0 (0)	3 (12)	1 (13)
Asthma *n*, %	5 (31)	2 (18)	0 (0)	2 (25)
Current use of asthma medication *n*, %	6 (40)	2 (18)	6 (24)	3 (43)

a *Exercise induced laryngeal obstruction; for details see text and Table 2*.

b *Number of participants providing data to number of participants in sub-group*.

### General Self-Rated Health

Most participants 61/70 (87%) rated their health as “good” or “very good,” and only one responded “poor.” There were no significant differences between the EILO subgroups (Tables 4, 5).

**Table 4:** Scores on patient reported outcome measures among participating adolescents with exercise induced laryngeal obstruction (EILO).

	***N***	**Mean (SD)**	**No. of items**	**Range**
General self-rated health^a^	70	3.24 (0.71)	1	1–4
HBSC-SCL^b^ Sum score^c^	69	9.55 (6.34)	8	0–25
Psychological sub score^d^	69	4.84 (3.45)	4	0–14
Somatic sub score^e^	68	4.77 (3.75)	4	0–13
SCARED^f^ Total score	69	1.84 (2.21)	5	0–10
GSE^g^	70	3.13 (0.42)	10	2.2–4.0

a *General health; possible range 1–4 (best)*.

b *Health Behavior in School-aged Children-Symptom Check List*.

c *Sum score; possible range 0–32*.

d *Psychological sub score; possible range 0–16*.

e *Somatic sub score; possible range 0–16*.

f *Screen for Child Anxiety Related Disorder; possible range 0–10*.

g *General self-efficacy; possible range 1–4*.

**Table 5:** Scores on patient reported outcome measures of 69 participants^a^, split by the four types of exercise induced laryngeal obstruction (EILO); for details see text and Table 2.

	**Normal (*n* = 19)**	**Predominantly glottic (*n* = 12)**	**Predominantly supraglottic (*n* = 29)**	**Combined glottic and supraglottic (*n* = 9)**
General health	3.1 (2.7–3.5)	3.4 (2.9–3.8)	3.3 (3.0–3.5)	3.1 (2.6–3.7)
HBSC-SCL^b^ Sum score	9.6 (6.3–12.8)	12.9 (7.9–17.9)	9.5 (7.4–11.6)	6.1 (2.5–9.8)
GSE^c^	3.1 (2.9–3.4)	3.1 (2.8–3.4)	3.1 (3.0–3.3)	3.2 (2.9–3.5)
SCARED^d^	1.9 (0.5–3.3)	2.1 (1.0–3.1)	1.6 (0.8–2.4)	1.9 (0.3–3.5)

*There were no significant group differences for any of the parameters (one way ANOVA)*.

*Figures are group mean values (95% confidence interval)*.

a *CLE scores missing for two responders*.

b *Health Behavior in School-aged Children – Symptom Check List*.

c *General self-efficacy*.

d *Screen for Child Anxiety Related Disorder*.

### Subjective Health Complaints (HBSC-SCL)

Headache and abdominal pain were the most common subjective somatic health complaints, with 28 (42%) and 20 (30%) reporting headache and abdominal pain weekly or more often, respectively. The most common subjective psychological health complaint was being irritable or in a bad mood, with 30 (43%) responding that this occurred weekly or more often. Mean HBSC-SCL score was 9.55, with responses ranging from 0 to 25 of the possible range 0–32. There were no significant differences between the four EILO subgroups (Tables 4, 5).

### Self-Efficacy

The overall mean score for GSE were 3.13, which is considered well within normal range. The scores did not differ between the four EILO subgroups (Tables 4, 5).

### Anxiety

The majority of the participants (*n* = 69) reported no symptoms of anxiety. The responses did not differ between the four EILO subgroups (Tables 4, 5).

## Discussion

The major finding from this study was that adolescents with EILO rated their health as good, with data for general health, subjective health complaints (psychological and somatic), self-efficacy, and anxiety symptoms similar to data obtained from comparable unselected populations. Moreover, these outcome variables were unrelated to EILO subtype and severity.

### Strengths and Weaknesses of the Study

A major strength of this study was that it approached all adolescents included in the EILO-registry between 2014 and 2016 at Haukeland University Hospital, which is a nationwide center for EILO and therefore receives referrals from a broad and representative selection of young people suffering from EILO. The idea was that this set-up should ensure a correspondingly broad and representative recruitment of EILO patients, producing outcome data with a high degree of generalizability. However, the rather disappointing rate of participation of 39% challenged this concept, and this is the obvious and major weakness of the study. Nonetheless, the finding that general health, subjective health complaints, self-efficacy, and anxiety were similarly distributed over EILO subtypes and severity certainly challenges the notion that these characteristics should be causally implicated in EILO. There were 82% female participants, which is approximately representative for the gender distribution of the EILO registry with 75% being females, and with previous studies reporting similarly high rates of EILO among females (1, 2). There was a high level of exercise frequency among the participants, which is also consistent with previous studies, reporting that EILO is more common in physically active adolescent and in athletes (1, 35). One may speculate if physically highly active adolescents who experience respiratory symptoms are more likely to seek medical advice than average adolescents with a more sedentary lifestyle. It was a limitation of the study that the participants received the questionnaires after they were diagnosed with EILO, and thus we do not know their status prior to this point in their life. It was also a limitation that we had no healthy controls matched to our participants, but instead had to compare our findings to normative data obtained in comparable large population studies. A co-existing asthma or use of asthma medication was relatively rare and also similarly distributed across the EILO sub-groups, and thus not likely to have influenced the major findings in significant ways.

### General Health and Health Behavior in School-Aged Children

The rate of subjective health complaints might seem high, but the findings were similar to data reported from a population based study of Norwegian adolescents (36). Moreover, a large international survey of pain in adolescents reported similarly high rates of health complaints, with more than 50 % reporting headache, and 49.8 % reporting abdominal pain weekly or more often (37). Thus, adolescents with EILO did not differ from otherwise comparable peers in terms of self-perceived health (25).

### General Self-Efficacy

The mean GSE score of 3.1 was comparable to data reported from a study of Norwegian otherwise comparable adolescents (38, 39). Self-efficacy determines how people feel, think, motivate themselves and behave in new and challenging situations and is considered an important feature in behavioral change and coping (40, 41). A Norwegian study of adolescents reported positive associations between GSE and health-related quality of life (39). Thus, the good (normal) GSE score observed in our EILO patients may help them cope with their condition.

### Anxiety

Anxiety symptoms measured the SCARED questionnaire were in line with data obtained in comparable reference populations (33). Moreover, as for the other parameters tested in this study, we found no gradients over EILO subtypes or severity. Particularly it was of interest, and somewhat surprising, that the level of anxiety for the glottic subtype of EILO was in line with the other EILO subgroups and with the data reported from a from a population based study (33). Two individuals had the highest possible score on the SCARED questionnaire (11); one of them had a normal laryngeal response to exercise (i.e., no EILO) and the other a predominantly glottic EILO.

The CLE scoring system has been questioned by some authors, and studies have reached somewhat variable conclusions regarding repeatability and precision (23, 42, 43). In order to avoid pitfalls in this area, only two highly experienced raters performed all scoring procedures, both having assessed CLE recordings from more than 2,000 individuals. Both raters reviewed the videos retrospectively and individually, but all in one session, solving disagreements subsequently by consensus. The CLE scoring system opened for studying if the outcomes that we addressed varied across EILO subtypes and EILO severity. We were particularly interested in the glottic subtype, as we have hypothesized that this EILO variant may resemble what was previously labeled exercise induced VCD. Moreover, it had seemed reasonable to hypothesis that if personality traits or psychological mechanisms were involved in the causal chain leading to EILO, this would be most evident in patients with the most severe subtype; i.e., in participants in whom *both* glottic and supraglottic scores were at least moderate or severe. However, we found no such tendencies; the confidence intervals for all the addressed parameters showed nearly full overlap in all subgroups. One way of interpreting this finding is that the addressed outcomes are not involved in the causal chain leading to EILO. However, due to our low rate of participation, this interpretation must obviously be tested in future studies. Moreover, we advise future studies to rate patients *before* they are tested and assigned the EILO diagnosis. We know that worldwide, too many of these patients are misdiagnosed as malingerers or as suffering from poorly controlled asthma, and therefore exposed to asthma medication with no effect (44, 45). To live for years in such situations conceivably challenges self-esteem and psychological well-being. Thus, a correct diagnosis relieves stress, and represents a “new beginning” for many patients.

## Conclusion

Our findings indicate good self-rated health and self-efficacy in adolescents with EILO, and no more anxiety symptoms than usually found in this age group. None of these outcome variables were associated with EILO subtype or severity. The findings question the notion that psychological problems are causally involved in EILO.

## Data Availability Statement

In accordance with the approvals granted for this study by The Regional Committee on Medical Research Ethics and The Norwegian Data Inspectorate, the data files are stored securely and in accordance with the Norwegian Law of Privacy Protection. The data file cannot be made publicly available as this might compromise the respondents' privacy. A subset of the data file with anonymized data can be made available to interested researchers upon reasonable request to Hege Clemm (hsyh@helse-bergen.no), providing Norwegian privacy legislation and GDPR are respected, and that permission is granted from The Norwegian Data Inspectorate and the data protection officer at Haukeland University Hospital.

## Ethics Statement

The studies involving human participants were reviewed and approved by The Regional Committee for Medical Research Ethics for Western Norway, Protocol no. 2017/299. Written informed consent to participate in this study was provided by the participants' legal guardian/next of kin.

## Author Contributions

MB collected the data, analyzed the data, drafted the initial manuscript, reviewed, and edited the manuscript. BV, MH, HC, OR, MV, and TH designed the data collection instruments. BV, TH, HC, and JD reviewed and edited the manuscript. All authors contributed to the article and approved the submitted version.

## Conflict of Interest

The authors declare that the research was conducted in the absence of any commercial or financial relationships that could be construed as a potential conflict of interest.
